# Changes in paraspinal muscles and facet joints after percutaneous endoscopic transforaminal lumbar interbody fusion for the treatment of lumbar spinal stenosis: A 3-year follow-up

**DOI:** 10.3389/fsurg.2022.1041105

**Published:** 2022-10-28

**Authors:** Daming Pang, Jincai Yang, Yong Hai, Zhexuan Fan, Haifeng Gao, Peng Yin

**Affiliations:** Department of Spine Surgery, Affiliated Beijing Chaoyang Hospital of Capital Medical University, Beijing, China

**Keywords:** transforaminal lumbar interbody fusion, endoscopy, paraspinal muscles atrophy, functional cross-sectional area, spondylolisthesis

## Abstract

**Objectives:**

This study investigates the changes in the paraspinal muscles of lumbar spinal stenosis patients after percutaneous endoscopic transforaminal lumbar interbody fusion (PE-TLIF).

**Methods:**

Thirty-three patients from Beijing Chaoyang Hospital who had L4/5 segment lumbar spinal stenosis between January, 2017 and January, 2019were included in this study. Patient-reported outcomes including the visual analog scale scores for back pain and leg pain (VAS-BP and VAS-LP, respectively) and the Oswestry disability index (ODI) scores at pre-operation and 1-week, 3-month, 12-month, and (at least) 3-year follow-up (the final follow-up) were evaluated. Computed tomography (CT) was performed at the 12-month follow-up, 24-month follow-up, and the final follow-up after surgery. Multifidus (MF) muscle functional cross-sectional area (FCSA) and fat infiltration (FI) were evaluated, and the degree of adjacent facet joint degeneration was evaluated using Pathria scores.

**Results:**

All patients underwent at least a 3-year follow-up period. The VAS-BP, VAS-LP, and ODI were significantly lower at 1-week, 3-month, 12-month, and 3-year follow-up than at pre-operation (*P* < 0.05). At the 3-year follow-up, no differences were found in FCSA and FI for any patient's MF muscle at the lower third of the vertebral body (L3) above the operation level (*P* > 0.05), and there was no statistical difference in the central plane of the L3/4 and L5/S1 vertebral facet joints at pre-operation, 12-month, 24-month, and 3-month follow-up (*P* > 0.05).

**Conclusions:**

PE-TLIF can provide satisfactory clinical outcomes for patients with lumbar spinal stenosis. Furthermore, the technique may also reduce the injury on the paravertebral muscles, especially the MF muscle, as well as on adjacent facet joints.

## Background

Since the 1990s, traditional open surgery has been widely used to treat Lumbar spinal stenosis (LSS) (1), a condition that affects 47.2% of people worldwide (2). However, paraspinal muscle atrophy, especially the multifidus (MF), and degeneration of the facet joints are frequently observed as a complication of the procedure during follow-up, due traditional open surgery's lack of protection of the paraspinal muscles and facet joints (3). Paraspinal muscles such as the MF and facet joints play an important role in maintaining the stability of lumbar vertebrae (4), and injuries to these structures can lead to chronic back pain (4–8). Therefore, finding effective treatments for stenosis that reduce the chance of injuries to these structures is of paramount importance.

Clinical trials have shown that minimally invasive interbody fusions are effective at reducing muscle injuries (9). In 2002, Foley and Lefkowitz introduced minimally invasive fusion technology for the first time, and the technique exhibited clear advantages in reduced trauma, wound size, and hospitalization time (10). However, the MIS-TLIF technique uses screws in a similar way to conventional open surgery, and this method can too often lead to the injury of the medial branch of the dorsal ramus, increasing the possibility of MF atrophy. As a result, surgeons have begun to attempt percutaneous screw fixation in lumbar surgery.

By applying a minimally invasive approach and developing spinal endoscopic techniques, we have developed some novel techniques for performing percutaneous endoscopic transforaminal lumbar interbody fusion. Furthermore, we have developed a guided superior articular process (SAP) resection device that can excise the articular processes precisely and reduce iatrogenic injury (11, 12). Thus, the objective of this study is to evaluate the changes in paraspinal muscles and facet joint degeneration after PE-TLIF and a follow-up period of at least 3 years in order to assess the clinical value of PE-TLIF in the treatment of lumbar spinal stenosis.

## Materials and methods

### Patient population

This retrospective study was performed at Beijing Chaoyang Hospital and included 33 patients who were diagnosed with lumbar spinal stenosis according their symptoms, clinical signs, and medical images and subsequently underwent PE-TLIF between January, 2017 and January, 2019. Inclusion criteria were as follows: (1) degenerative instability on the L4/5 level and LSS; (2) receipt of PE-TLIF treatment; and (3) a follow-up period of at least 3 years. Exclusion criteria were: (1) receipt of previous spinal surgery and (2) suffering from infection, trauma, or spondylolisthesis. This study was approved by the Ethics Committee of Beijing Chaoyang Hospital.

### Surgical technique

The specific procedure is detailed in our previous research (12).

### Data collection

**Figure 1:**
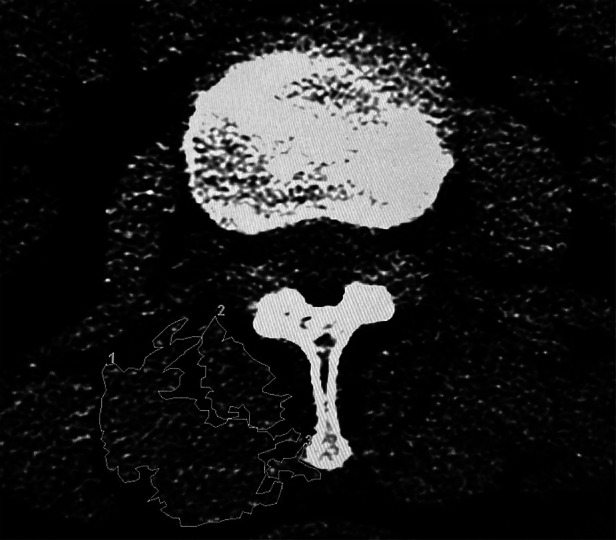
Measurement of the MF FCSA in an atrophied muscle.

In order to examine the changes in paraspinal muscles and facet joints degeneration after PE-TLIF, we obtained MF functional cross-sectional area (FCSA) (13) and fat infiltration (FI) measurements from axial CT axial images at the lower third of the vertebral body (L3) above the operation level (L4/5) before surgery and 12 months, 24 months, and 3 years (or final follow-up if longer than 3 years) after surgery to avoid any artifacts produced by the screws themselves. In addition, to prevent interference from the nearby fat, bony structures, and other soft tissues, we measured the MF FCSA and FI using purpose-built software from GE Healthcare (United States) according to the manufacturer's selection method for muscle regions of interest (ROI) (Figure 1) (14). The FI rate was graded according to the degree to which MF muscle was replaced by adipose tissue: “0” for estimates of normal or no obvious FI within the muscle, “1” for <10% FI, “2” for 10%–50% FI, and “3” for >50% FI. We obtained the total segmental value for FI by summing the left and right values.

The central planes of the L3/4 and L5/S1 vertebral facet joints were qualitatively evaluated using the Pathria grading system in axial scanning CT imaging (15), and once again we obtained the total segmental scores for each level by summing the left and right Pathria scores for that level. Clinical effects, including the visual analog scale scores (VAS) for back pain and leg pain (VAS-LBP and VAS-LP, respectively) and the Oswestry disability index (ODI), were evaluated at pre-operation, and at the 1-week, 3-month, 12-month and final follow-ups.

### Statistical analyses

All data were analyzed using SPSS 21.0 software. We used the Friedman rank-sum test for nominal data and repeated measures analysis of variance for continuous data in order to test MF functional cross-sectional area. For each test, we considered a *P* < 0.05 to indicate a statistically significant result.

## Results

### Patient demographics

**Figure 2:**
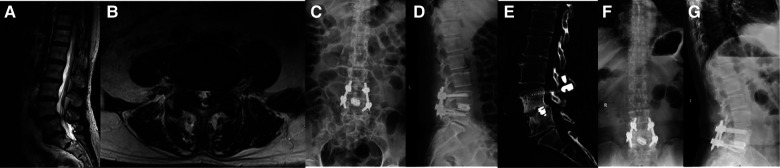
A 69-year-old female. Preoperative VAS-LBP: 6; Preoperative VAS-LP: 8; Preoperative ODI: 70%. (**A, B**) A L4/5 spinal stenosis identified in the preoperative MRI. (**C, D**) A good implantation position shown by x-rays taken a week after the operation. (**E**) At 12 months after the surgery, a CT scan image revealed a standard lumbar fusion. (**F, G**) The final x-ray images indicated that the implantation occurred in a good position.

A total of 33 patients were included in this study. There were 13 male and 20 female patients, and the mean age of patients was 61.0 ± 8.9 years (range, 45–82 years). All patients received a follow-up period of at least 3 years, and the average follow-up period was 41.7 ± 3.5 months. The mean body mass index (BMI) was 23.5 ± 3.9 kg/m^2^, and the average operation time was 208.7 ± 28.5 min. Mean blood loss from the operation was 138.2 ± 83.5 ml, and the average postoperative rest time for each patient was 17.9 ± 2.2 days (see Table 1 and Figure 2).

**Table 1:** Characteristics of patients.

Characteristics	*n*
Gender	
Male	13
Female	20
Age (years)	59.0 ± 8.9
BMI (kg/m^2^)	23.5 ± 3.9
Average incision length (cm)	8.7 ± 2.5
Operation time (min)	208.7 ± 28.5
Intraoperative blood loss (ml)	138.2 ± 83.5
Postoperative rest time (days)	17.9 ± 2.2
Follow-up period (months)	41.7 ± 3.5

### Postoperative outcomes

The ODI score decreased from 62% (56,65) at pre-operation to 24% (20,30) at 3-month follow-up, 12% (9.5,16.5) at 12-month follow-up, and 8% (4,15.5) at final follow-up. The VAS-LBP decreased from 7 (7,8) at pre-operation to 3 (2,3) at 1-week follow-up, 1 (1,2) at 3-month follow-up, 1 (0,2) at 12-month follow-up, and 1 (0,1) at final follow-up. Similarly, the VAS-LP decreased from 6 (5,7) at pre-operation to 2 (1,3) at 1-week follow-up, 1 (1,2) at 3-month follow-up, 1 (0,2) at 12-month follow-up, and 0 (0,1) at final follow-up (Table 2). Compared to the preoperative FCSA of the MF, the postoperative FCSA of the MF for any follow-up stage was not statistically different (*P* > 0.05). Furthermore, the median preoperative MF muscle FI was 2, the median 12-month postoperative MF muscle FI was 3, the median 24-month postoperative MF muscle FI was 3, and the median final follow-up MF muscle FI was 3. None of these differences were statistically different from 0 (*P* > 0.05) (Table 3).

**Table 2:** Comparison of indicators related to efficacy evaluation before and after PE-TLIF.

*n* = 33	VAS-LBP	VAS-LP	ODI (%)
Pre-operation	7 (7,8)	6 (5,7)	62 (56,65)
Post-1 w	3 (2,3)*	2 (1,3)*	–
Post-3 m	1 (1,2)*	1 (1,2)*	24 (20,30)*
Post-12 m	1 (0,2)*	1 (0,1)*	12 (9.5,16.5)*
Final follow-up	1 (0,1)*	0 (0,1)*	8 (4,15.5)*

Note. *Compared to pre-operation, *P *< 0*.*05.

**Table 3:** Preoperative and postoperative paraspinal muscle parameters.

	Preoperative	Post-12 m	Post-24 m	Final follow-up
FCSA of the MF	(568.09 ± 49.82)	(557.12 ± 51.31)*	(558.55 ± 53.37)*	(537.51 ± 55.11)*
FI of the MF	2 (2,3)	3 (2,3)*	3 (2,3)*	3 (3,3)*

Note. *Compared to pre-operation, *P *> 0*.*05.

The postoperative upper segment facet joint scores at 12 months and 24 months were also not statistically different compared to pre-operation (*P* > 0.05), and compared to the preoperative lower segment facet joint score, the postoperative lower segment facet joint scores at 12 months and 24 months were not statistically different either (*P* > 0.05) (Table 4). Finally, intervertebral fusion was completed in all patients after 12 months, according to the Bridwell criteria (16), Grade I in 13 cases, Grade II in 16 cases, and Grade III in 4cases.

**Table 4:** Preoperative and postoperative scores of the segment facet joints.

	Preoperative	Post-12 m	Post-24 m	Final follow-up
USFJ	5 (4,6)	5 (4,6)*	5 (4,5)*	5 (4,6)*
LSFJ	5 (4,6)	5 (4,5)*	5 (4,6)*	5 (4,7)*

Note. *Compared to pre-operation, *P *> 0*.*05.

## Discussion

The focus of our study was on the effects of PE-TLIF for single segment LSS on the MF and facet joints. During the follow-up period for this procedure, we found that use of the PE-TLIF technique provided adequate protection for the MF and adjacent facet joints. Additionally, the PE-TLIF surgery significantly improved the ODI and VAS for all patients, indicating that the clinical symptoms of the patients had been effectively relieved.

The MF is the most important stabilizing muscle of the spine and is located in the deepest part of the spinal column. During spinal movement, two-thirds of stabilizing stiffness is provided by the MF. In lumbar surgery, it is necessary to protect paraspinal muscles.

The traditional posterior surgery has great trauma, which causes direct damage to the paraspinal muscles in the process of muscle stripping. In addition, Tsutsumimoto et al. (17) believe that the continuous stretch of the paraspinal muscle caused by the retractor increases the pressure of the paraspinal muscle and affects the blood perfusion of the capillaries of the paraspinal muscle in traditional open surgery. This ischemic change of paraspinal muscles will eventually lead to functional changes of paraspinal muscles and muscle atrophy. The PE-TLIF technology we have developed has the advantages of smaller incision size and reduced paravertebral muscle dissection, which reduces the direct injury to the paraspinal muscle and the influence on the blood circulation of the paraspinal muscle. Moreover, through the analysis of the morphological changes of the multifidus in MRI after the injury of the the medial branch of the dorsal ramus after operation, it is considered that the muscle atrophy after lumbar surgery is related to the iatrogenic injury of the medial branch of the dorsal ramus (18–21). According to anatomy, it was found that the MF was innervated only by the medial branch. Traditional posterior open surgery increases the probability of injury of this nerve and the possibility of denervated atrophy of MF. In order to reduce the risk of the nerve injury, different from the traditional posterior lumbar interbody fusion and minimally invasive fusion technique, we chose to use the percutaneous method. According to Regev et al. (22), percutaneous screw placement can reduce the incidence of indirect injury to the medial branch nerve from 84% to 20%.

In order to evaluate the changes of MF after PE-TLIF, we compared the FCSA and FI rate of MF at pre-operation, 12-month follow-up, 24-month follow-up, and at final follow-up. Some studies have shown that the decrease of muscle volume and the increase of fat deposition are the main characteristics of paraspinal muscle degeneration (23, 24). Kang et al. confirmed by MRI that the degree of paraspinal muscle degeneration can be reflected by the decrease of paraspinal muscle FCSA and FI (25). In our study, we used FCSA and FI to assess the degree of MF atrophy by CT axial images. FCSA assessed by CT has high intraclass correlation with MRI according to Hu et al. (26). Due to interference from the metal artifacts, however, the MF had to be measured in selected axial images (27). The results showed that the FI rate of the MF muscles did not change significantly at 12 months, 24 months, or the final follow-up after surgery. Some studies have reported that the function of the MF can be affected after operation (28, 29), but in this study we found no difference in the FCSA of the MF at 12 months, 24 months, or the final follow-up after PE-TLIF.

In order to help us perform this novel procedure, we have invented a cannula with a hook-shaped front. This tool can effectively remove part of the articular process and protect local tissue. By reducing intraoperative trauma, the impact on adjacent segments can be reduced. The adjacent segmental facet joint scores of the 33 patients in this study were not statistically different from their preoperative scores. In our study, all the patients achieved satisfactory clinical results, and their postoperative lumbar pain was significantly reduced. No obvious degeneration of adjacent facet joint was found at least 3 years after operation, but for long-term results, it is necessary to analyze the effects of degeneration and operation on adjacent segments, so as to evaluate the results of PE-TLIF. However, this study has several limitations. First, the study was retrospective and single-center in design, and it was neither randomized nor controlled. Second, the sample size was small. Finally, we couldn't estimate the effects of our procedure on the muscles at the fusion level due to the interference from the metal artifacts.

## Conclusion

Our novel PE-TLIF can provide satisfactory clinical outcomes for patients with lumbar spinal stenosis. By the avoiding direct injury to the paraspinal muscle and the traction of the paraspinal muscle, and reducing the injury probability of the medial dorsal branch, Our PE-TLIF can adequately protect the MF and reduce the degeneration of the adjacent facet joints.

## Data Availability

The original contributions presented in the study are included in the article/Supplementary Material, further inquiries can be directed to the corresponding author/s.

## References

[1] Farrokhi MR Yadollahikhales G Gholami M Mousavi SR Mesbahi AR Asadi-Pooya AA. Clinical outcomes of posterolateral fusion vs. Posterior lumbar interbody fusion in patients with lumbar spinal stenosis and degenerative instability. Pain Phy. (2018) 21:383–406. 10.36076/ppj.2018.4.383. 30045595

[2] Cole AA. Fusion for lumbar spinal stenosis? BMJ. (2016) 353:i3145. 10.1136/bmj.i3145. 27287461

[3] Fan SW Hu ZJ Fang XQ Zhao FD Huang Y Yu HJ. Comparison of paraspinal muscle injury in one-level lumbar posterior inter-body fusion: modified minimally invasive and traditional open approaches. Orthop Surg. (2010) 2:194–200. 10.1111/j.1757-7861.2010.00086.x. 22009948 PMC6583124

[4] Freeman MD Woodham MA Woodham AW. The role of the lumbar multifidus in chronic low back pain: a review. PM R. (2010) 2:142–6, 1-167. 10.1016/j.pmrj.2009.11.006. 20193941

[5] Lee JC Cha JG Kim Y Kim YI Shin BJ. Quantitative analysis of back muscle degeneration in the patients with the degenerative lumbar flat back using a digital image analysis: comparison with the normal controls. Spine (Phila Pa 1976). (2008) 33:318–25. 10.1097/BRS.0b013e318162458f. 18303466

[6] Shafaq N Suzuki A Matsumura A Terai H Toyoda H Yasuda H Asymmetric degeneration of paravertebral muscles in patients with degenerative lumbar scoliosis. Spine (Phila Pa 1976). (2012) 37:1398–406. 10.1097/BRS.0b013e31824c767e. 22322373

[7] Sihvonen T Herno A Paljärvi L Airaksinen O Partanen J Tapaninaho A. Local denervation atrophy of paraspinal muscles in postoperative failed back syndrome. Spine (Phila Pa 1976). (1993) 18:575–81. 10.1097/00007632-199304000-00009. 8484148

[8] Cho SM Kim SH Ha SK Kim SD Lim DJ Cha J Paraspinal muscle changes after single-level posterior lumbar fusion: volumetric analyses and literature review. BMC Musculoskelet Disord. (2020) 21:73. 10.1186/s12891-020-3104-0. 32024500 PMC7003350

[9] Schwender JD Holly LT Rouben DP Foley KT. Minimally invasive transforaminal lumbar interbody fusion (TLIF): technical feasibility and initial results. J Spinal Disord Tech. (2005) 18(Suppl):S1–6. 10.1097/01.bsd.0000132291.50455.d0. 15699793

[10] Foley KT Lefkowitz MA. Advances in minimally invasive spine surgery. Clin Neurosurg. (2002) 49:499–517. 12506566

[11] Yang J Liu C Hai Y Yin P Zhou L Zhang Y Percutaneous endoscopic transforaminal lumbar interbody fusion for the treatment of lumbar spinal stenosis: preliminary report of seven cases with 12-month follow-up. Biomed Res Int. (2019) 2019:3091459. 10.1155/2019/3091459. 31019966 PMC6451828

[12] Yin P Gao H Zhou L Pang D Hai Y Yang J. Enhanced recovery after an innovative percutaneous endoscopic transforaminal lumbar interbody fusion for the treatment of lumbar spinal stenosis: a prospective observational study. Pain Res Manag. (2021) 2021:7921662. 10.1155/2021/7921662. 34966474 PMC8712142

[13] Yoo JS Min SH Yoon SH Hwang CH. Paraspinal muscle changes of unilateral multilevel minimally invasive transforaminal interbody fusion. J Orthop Surg Res. (2014) 9:130. 10.1186/s13018-014-0130-3. 25499767 PMC4269953

[14] Crawford RJ Cornwall J Abbott R Elliott JM. Manually defining regions of interest when quantifying paravertebral muscles fatty infiltration from axial magnetic resonance imaging: a proposed method for the lumbar spine with anatomical cross-reference. BMC Musculoskelet Disord. (2017) 18:25. 10.1186/s12891-016-1378-z. 28103921 PMC5247810

[15] Pathria M Sartoris DJ Resnick D. Osteoarthritis of the facet joints: accuracy of oblique radiographic assessment. Radiol. (1987) 164:227–30. 10.1148/radiology.164.1.3588910. 3588910

[16] Bridwell KH Lenke LG McEnery KW Baldus C Blanke K. Anterior fresh frozen structural allografts in the thoracic and lumbar spine. Do they work if combined with posterior fusion and instrumentation in adult patients with kyphosis or anterior column defects? Spine (Phila Pa 1976). (1995) 20:1410–8. 10.1097/00007632-199506020-00014. 7676341

[17] Tsutsumimoto T Shimogata M Ohta H Misawa H. Mini-open versus conventional open posterior lumbar interbody fusion for the treatment of lumbar degenerative spondylolisthesis: comparison of paraspinal muscle damage and slip reduction. Spine (Phila Pa 1976). (2009) 34:1923–8. 10.1097/BRS.0b013e3181a9d28e. 19652636

[18] Hultman G Nordin M Saraste H Ohlsèn H. Body composition, endurance, strength, cross-sectional area, and density of MM erector spinae in men with and without low back pain. J Spinal Disord. (1993) 6:114–23. 10.1097/00002517-199304000-00004. 8504222

[19] Barker KL Shamley DR Jackson D. Changes in the cross-sectional area of multifidus and psoas in patients with unilateral back pain: the relationship to pain and disability. Spine (Phila Pa 1976). (2004) 29:E515–9. 10.1097/01.brs.0000144405.11661.eb. 15543053

[20] Hansen L de Zee M Rasmussen J Andersen TB Wong C Simonsen EB. Anatomy and biomechanics of the back muscles in the lumbar spine with reference to biomechanical modeling. Spine (Phila Pa 1976). (2006) 31:1888–99. 10.1097/01.brs.0000229232.66090.58. 16924205

[21] Tsutsumimoto T Shimogata M Ohta H Misawa H. Mini-open versus conventional open posterior lumbar interbody fusion for the treatment of lumbar degenerative spondylolisthesis: comparison of paraspinal muscle damage and slip reduction. Spine (Phila Pa 1976). (2009) 34:1923–8. 10.1097/BRS.0b013e3181a9d28e. 19652636

[22] Regev GJ Lee YP Taylor WR Garfin SR Kim CW. Nerve injury to the posterior rami medial branch during the insertion of pedicle screws: comparison of mini-open versus percutaneous pedicle screw insertion techniques. Spine (Phila Pa 1976). (2009) 34:1239–42. 10.1097/BRS.0b013e31819e2c5c. 19444073

[23] Fan SW Hu ZJ Fang XQ Zhao FD Huang Y Yu HJ. Comparison of paraspinal muscle injury in one-level lumbar posterior inter-body fusion: modified minimally invasive and traditional open approaches. Orthop Surg. (2010) 2:194–200. 10.1111/j.1757-7861.2010.00086.x. 22009948 PMC6583124

[24] Kameyama K Ohba T Endo T Katsu M Koji F Kensuke K Radiological assessment of postoperative paraspinal muscle changes after lumbar interbody fusion with or without minimally invasive techniques. Global Spine J. (2021):302288682. 10.1177/2192568221994794. PMC9972276 33657897

[25] Kang CH Shin MJ Kim SM Lee SH Lee CS. MRI Of paraspinal muscles in lumbar degenerative kyphosis patients and control patients with chronic low back pain. Clin Radiol. (2007) 62:479–86. 10.1016/j.crad.2006.12.002. 17398274

[26] Hu ZJ He J Zhao FD Fang XQ Zhou LN Fan SW. An assessment of the intra- and inter-reliability of the lumbar paraspinal muscle parameters using CT scan and magnetic resonance imaging. Spine (Phila Pa 1976). (2011) 36:E868–74. 10.1097/BRS.0b013e3181ef6b51. 21224757

[27] He W He D Sun Y Xing Y Liu M Wen J Quantitative analysis of paraspinal muscle atrophy after oblique lateral interbody fusion alone vs. Combined with percutaneous pedicle screw fixation in patients with spondylolisthesis. BMC Musculoskelet Disord. (2020) 21:30. 10.1186/s12891-020-3051-9. 31937277 PMC6961348

[28] Fan SW Hu ZJ Fang XQ Zhao FD Huang Y Yu HJ. Comparison of paraspinal muscle injury in one-level lumbar posterior inter-body fusion: modified minimally invasive and traditional open approaches. Orthop Surg. (2010) 2:194–200. 10.1111/j.1757-7861.2010.00086.x. 22009948 PMC6583124

[29] Zhu HF Wang GL Zhou ZJ Fan SW. Prospective study of long-term effect between multifidus muscle bundle and conventional open approach in one-level posterior lumbar interbody fusion. Orthop Surg. (2018) 10:296–305. 10.1111/os.12402. 30402963 PMC6594533

